# Nonclinical human neural new approach methodologies (NAMs): Electrophysiological assessment of opioid agonist and antagonist combination

**DOI:** 10.1016/j.namjnl.2025.100064

**Published:** 2025-10-22

**Authors:** Carlos Serna, Bhavya Bhardwaj, Tromondae K. Feaster, Ksenia Blinova

**Affiliations:** Division of Applied Regulatory Science, Office of Clinical Pharmacology, Office of Translational Sciences, Center for Drug Evaluation and Research, U.S. Food and Drug Administration, Silver Spring, MD, United States

**Keywords:** New approach methodologies (NAMs), Human induced pluripotent stem cells (hiPSCs), Multielectrode array (MEA), Opioid, Drug combination

## Abstract

**Background and Purpose:**

New approach methodologies (NAMs) including microphysiological systems in combination with human induced pluripotent stem cell (hiPSC)-derived neural cells and multielectrode array (MEA) have demonstrated utility for evaluating electrophysiological effects of CNS active compounds including those with potential seizurogenic liability. Here, we extend a neural NAM assay to assessment of an opioid agonist and reversal agent.

**Methods:**

An in vitro human NAM composed of hiPSC-neurons and hiPSC-astrocytes coupled with MEA platform was used to assess spontaneous neural electrophysiology. Neural cells were cultured on MEA plates then treated with varying concentrations of the µ-opioid receptor agonist DAMGO (D-Ala(2)-mephe(4)-gly-ol(5))enkephalin). Following DAMGO treatment, the opioid antagonist naloxone [10 µM] was added to each well to evaluate phenotypic reversal.

**Results:**

The opioid agonist DAMGO modulated neural electrophysiological activity in a concentration-dependent manner relative to vehicle control. These changes were observed across neural parameters relating to overall neural activity, single electrode burst, network burst, and synchronicity. These effects were reversed by the opioid antagonist naloxone. Moreover, DAMGO treatment disrupted higher order baseline neural patterns. Finally, subpopulation analysis revealed divergent opioid-induced higher order network response influenced by baseline network activity.

**Conclusions:**

These findings demonstrate the hiPSC neural NAM can characterize human network electrical activity at baseline and following exposure to an opioid agonist and antagonist pair. Future studies will investigate additional opioids and reversal agents, as well as other MEA metrics and activity patterns that may best describe concentration- and time-dependent changes in neural activity.

## Highlights

• hiPSC neural NAM responds to opioids in a concentration-dependent manner.
• The observed changes are reversed by the opioid antagonist naloxone.
• DAMGO treatment disrupts higher order synaptic network connectivity.
• DAMGO-induced network burst effects are mediated by the NAMs baseline properties.
• hiPSC neural MEA assay develops robust baseline neural network patterns.

## Graphical abstract

**Figure:**
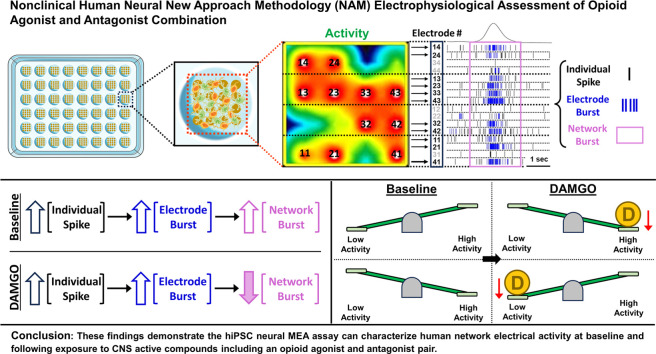

## 1 Introduction

To improve pharmacological safety assessment, regulatory initiatives such as the FDA Modernization Act 2.0 and Roadmap to Reducing Animal Testing in Preclinical Safety Studies have increased the adoption of new approach methodologies (NAMs) focused on reducing reliance on animal testing while advancing the development of human-relevant models (Congress, 2022; FDA, 2025). For examining neural networks, multielectrode array (MEA) technology has emerged as a powerful in vitro NAM platform capable of characterizing the electrophysiological effects of pharmaceutical compounds on complex cell constructs. Using embedded electrodes within the culture plate, these systems provide a high-throughput format and real time monitoring of electrical activity within neural co-culture models that can identify drug-induced changes in neural function and connectivity (Belair et al., 2025). In terms of translational relevance, integration of human induced pluripotent stem cell (hiPSC)-derived neural cells with the MEA platform provides the advantage of generating human-specific cellular responses. Previously, MEA-based models utilizing hiPSC neural cells have been established for evaluating drug-induced seizurogenic liabilities (Belair et al., 2025; Rockley et al., 2023; A.M. Tukker et al., 2020; Zhai et al., 2023), demonstrating their potential in predicting adverse neurological effects and supporting their use in regulatory decision-making and safety pharmacology studies for central nervous system (CNS) active compounds (Grainger et al., 2018; Zhao et al., 2024).

As one of the most challenging public health crises of our time, efforts to address the ongoing opioid epidemic require novel approaches to better understand the underlying therapeutic mechanisms and safety profiles of opioid compounds and antagonists, particularly in the context of CNS interactions. Though much of our foundational knowledge of the CNS response to opioid pharmacology has advanced greatly through nonclinical animal studies (Gumnit et al., 2022; Miner et al., 2021; Welsch et al., 2023), a shift in the paradigm now places emphasis on developing human-relevant in vitro models capable of assessing the electrophysiological effects of various opioids and their reversal agents. This is particularly important for misused opioid pain medications and illicit synthetic opioids, such as fentanyl. Likewise, detailed evaluation of the effects of opioid overdose reversal agents including naloxone, will be useful to determine concentrations needed to reverse opioid-induced toxicity and potentially inform dose requirements and recommendations.

In the present study, we leverage the validated framework of hiPSC-derived neural cells and MEA technology routinely used for seizure assessment into a comprehensive approach for rapidly identifying and assessing electrophysiological signatures of opioid related activation and antagonism (Fan et al., 2019). Specifically, we investigate the modulation of neural electrical activity induced by [D-Ala2, N-MePhe4, Gly-ol]-enkephalin (DAMGO), a selective μ-opioid receptor agonist, and naloxone, a competitive opioid receptor antagonist by examining the effects on spontaneous and synchronous bursting patterns representative of neuronal network communication. Our findings demonstrate that a nonclinical neural NAM can mimic key CNS physiological responses following acute opioid modulation. Consequently, this work establishes an in vitro platform to assess various combinations of opioid polypharmacy in human neurons, ultimately reducing the dependence on animal studies.

## 2 Methods

### 2.1 Cell culture

Cryopreserved glutamatergic-enriched neurons, iCell GlutaNeurons 01279, Catalog Number: R1034, Lot Number: 107224 (79 % glutamatergic neurons, 21 % GABAergic neurons), and specialized glial cells, iCell Astrocytes 2.0 01279, Catalog Number: R1240 (Fujifilm Cellular Dynamic, Inc. Madison, WI, USA) derived from human induced pluripotent stem cells (hiPSCs) were thawed and plated, as previously described in (Rockley et al., 2023; Zhai et al., 2023) accordance to the manufacturer’s instructions. The hiPSC line used in this study was reprogrammed from a blood donor sample, isolated from an apparently “healthy” normal Caucasian male, aged 50 to 59 years old. In brief, the neural cells were thawed and carefully suspended through dropwise addition of complete BrainPhys medium (cBPM) composed of BrainPhys Neuronal Medium (STEMCELL Technologies), 2 % iCell Neural Supplement B (Fujifilm Cellular Dynamic, Inc.), 1 % iCell Nervous System Supplement (Fujifilm Cellular Dynamic, Inc.), 1 % N-2 Supplement (Thermo Fisher Scientific), 0.1 % laminin (1 mg/mL, Fujifilm Wako Pure Chemical Corp.), and 1 % Penicillin-Streptomycin (Thermo Fisher Scientific). Following centrifugation, the cells were then resuspended in dotting medium (cBPM with 10 % laminin) to obtain densities of 15×10^6^ iCell GlutaNeurons/mL and 6.7 × 10^6^ iCell Astrocytes/mL. In preparation for plating onto 48-well multielectrode array plates (Axion BioSystems, Catalog Number: M768-tMEA-48 W), both cell types were combined into a “master mix” containing a 6:1 neuron to astrocyte ratio. 11 µL droplets from this suspension were then dispensed over each electrode region pre-coated with 0.1 % Polyethylenimine solution (Sigma Aldrich) resulting in a deposition of 120,000 neurons and 20,000 astrocytes per well. To prevent evaporation and sustain environmental conditions, 3 mL of sterile water was added to the area surrounding the wells. After allowing 1 hour for cell adherence, 300 µL of cBPM was then added in two phases to each well after which the plate was returned to incubation at 37ºC, 5 % CO_2_. A 50 % medium change was then performed every 2 to 3 days while sustaining peripheral water levels. The neural co-culture was maintained for 21 to 23 days to allow for the formation of functional networks prior to testing compounds of interest.

### 2.2 Compound treatment

On the day of the assay, complete BrainPhys medium was equilibrated to room temperature, and 150 μL of spent medium from each MEA plate well was transferred to a sterile culture plate, then mixed with an equal volume of fresh complete BrainPhys medium to create a 50:50 spent/fresh medium mixture (300 μL total volume). The remaining spent medium was aspirated from the MEA plate, and 250 μL of the medium mixture was added to each well and allowed to incubate for at least 2 to 4 h at 37 °C with 5 % CO₂ prior to conducting the experiment. DAMGO (Sigma Aldrich) and naloxone (Sigma Aldrich) were prepared as stock solutions in DMSO (Sigma Aldrich) and diluted with complete BrainPhys medium to 6X the target concentrations in a separate compound plate and equilibrated to 37 °C for at least 15 min. 50 μL of the 6X compound solutions were then transferred to the MEA plate to achieve final concentrations of 2.5, 5, 10, 20 μM for DAMGO in a final volume of 300 μL. As to minimize alterations to the various DAMGO concentrations, 31X naloxone (i.e., 10 μL) in medium was added to the wells containing DAMGO for a total final volume of 310 μL resulting in a 10 μM concentration of naloxone 2 h following the addition of DAMGO. Vehicle control wells received equivalent volume of DMSO in medium (<0.1 % final concentration). Data recordings were then conducted at baseline and subsequent intervals following the addition of both compound treatments.

### 2.3 MEA recordings

Neuronal activity from the MEA plate containing hiPSC derived neurons and astrocytes was collected using the AxIS Navigator 3.1.2 software through the Maestro Pro Platform (Axion BioSystems). In brief, action potentials generated by the co-culture were recorded on days 5, 14, 21, and 22 following cell plating (Day 0). On data acquisition days, a 50 % medium change was performed on the MEA plate and left to incubate for 2 to 4 h at 37º C with 5 % CO₂. Prior to recording, the plate was moved from the incubator into the Maestro Pro system and allowed at least 10 min to reach environmental equilibrium. At this stage, baseline activity was then recorded for a duration of 600 s. On the day of the assay, following the acquisition of baseline activity, the co-culture was first treated with 2.5, 5, 10, 20 μM DAMGO then with 10 μM of naloxone as described in Section 2.2 *Compound Treatment*. Neuronal activity was recorded in 600 s intervals 90 min following the addition of DAMGO and 30 min after introducing the reversal agent naloxone.

### 2.4 Data analysis

Continuous voltage streams were captured by the AxIS Navigator 3.1.2 software (Axion BioSystems) using the spontaneous neural real-time configuration with added spike and electrode burst detection processors. The adaptive threshold crossing method was selected for spike detection and set to 6 x standard deviations. For single electrode burst detection, the inter-spike interval (ISI) threshold method was selected with the maximum ISI set to 100 ms and the minimum number of spikes set to 5. Data were then batch processed to generate AxIS spike (.spk) files then loaded onto the Neural Metric Tool for further analysis. While settings for electrode burst detection were left unchanged, the envelope algorithm was selected for network burst detection with a threshold factor of 1.25, minimum inter-burst interval of 100 ms, burst inclusion of 75 %, and minimum number of electrodes set to 35 %. Post-processed data was broadly categorized into spiking, single-electrode bursting, and network bursting metrics each with their own set of sub-parameters using a comparable analytical approach others have reported (Black et al., 2024; Kapucu et al., 2022; Pre et al., 2022; Seki et al., 2025). Acquisition criteria were selected with modifications to previously described criteria including exclusion of wells with ≤ 0.5 network burst per minute (Mossink et al., 2021) and ≤ 35 % active electrodes per well (Zhai et al., 2023). The double delta (ΔΔ) was determined by first calculating the difference between treated wells and their corresponding baseline values. These values are then compared to the vehicle control (DMSO) at the respective treatment timepoint (Blinova et al., 2018; Patel et al., 2019). Principal component analysis (PCA) was used to reduce dimensionality and analyze MEA parameters. PCA was performed in GraphPad Prism 10 and plotted against the two major principal components. Baseline subtracted (Δ) data obtained from MEA parameters was used for the PCA.

### 2.5 Statistical analysis

All statistical analyses were performed using GraphPad Prism 10. Data are presented as a mean ± standard error of the mean. Comparisons between any two data sets were conducted using paired and unpaired two-tailed *t*-tests for normally distributed data. The Wilcoxon Matched Pairs Signed-Rank Test or the Mann-Whitney U Test (unpaired) were used for analyzing non-normally distributed data. Control and treatment groups were compared to one another using one-way ANOVA or the Kruskal Wallis Test followed by post-hoc Tukey’s HSD and Dunn’s Multiple Comparisons tests, for normal and non-normal data, respectively. Normality was evaluated with a Shapiro - Wilk test. Results were considered statistically significant if the p value was <0.05.

## 3 Results

### 3.1 Nonclinical neural NAM

**Fig. 1:**
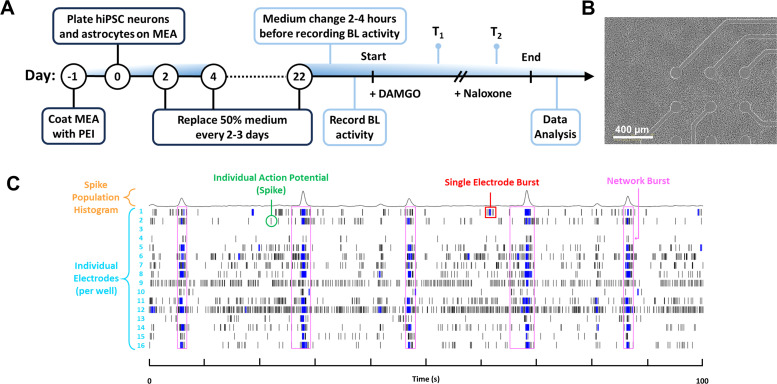
Nonclinical Neural NAM. A) Schematic of neural co-culture development and experimental timeline indicating compound treatment and data acquisition (BL, T1, T2). B) brightfield image (10x magnification, scale bar 400 µm) of neural co-culture at Day 23. C) Example Raster Plot from 100 s recording showing neural activity from a single well. Electrical activity is collected from 16 electrodes (cyan) capturing spikes (green), electrode bursts (red), and network bursts (pink). Spiking activity occurring in unison across the 16 electrodes is used to generate a population histogram displayed at the top of the raster plot (orange). BL=Baseline, *T*=Timepoint.

A neural NAM assay composed of commercially available hiPSC-derived excitatory glutamatergic neurons, inhibitory GABAergic neurons, and astrocytes was co-cultured for 22 days on MEA plates (Fig. 1) (Table 1). To elucidate how the electrophysiological metrics of the neural hiPSC MEA co-culture developed in vitro, we performed a longitudinal assessment of the network activity over time (Supplemental Figure 1). Consistent with previous reports, we found the spontaneous synchronous neural electrical activity increased with time in culture (A.M. Tukker et al., 2020). As such, there was a significant time-dependent increase in individual spike, single electrode burst, and global network burst metrics spanning across the 22 days (Supplemental Figure 1 B-D). Herein, we quantified the effects of opioid agonist DAMGO and opioid antagonist naloxone on hiPSC based neuronal electrophysiological activity relative to that of baseline and time-matched vehicle control to determine if the neural NAM could elucidate opioid-induced effects in vitro.

**Table 1:** Baseline hiPSC neural MEA electrophysiological properties day 22.

Parameter	Mean ± SEM
Activity Metrics	
Number of Spikes	14,206 ± 645
Number of Active Electrodes	11.16 ± 0.3
Weighted Mean Firing Rate (Hz)	1.48 ± 0.1
Electrode Burst Metrics	
Number of Electrode Bursts	411.57 ± 23.6
Number Burst per minute	41.16 ± 2.4
Number of Bursting Electrodes	9.46 ± 0.3
Burst Duration (*sec*)	0.33 ± 0.01
Number of Spikes per Burst	11.97 ± 0.5
Network Burst Metrics	
Number of Network Bursts	25.7 ± 1.7
Network Burst per minute	2.57 ± 0.2
Network Burst Duration (*sec*)	5.12 ± 0.4
Number of Spikes per Network Burst	295.15 ± 21.6
Mean ISI within Network Burst (*sec*)	0.022 ± 0.0014
Median ISI within Network Burst (*sec*)	0.014 ± 0.0010
Median/Mean ISI within Network Burst	0.55 ± 0.013
Number of Electrodes Participating in a Network Burst	10.75 ± 0.3
Number of Spikes per Network Burst per Channel	25.30 ± 1.48
Network Burst Percentage	42.12 ± 1.42
Network IBI Coefficient of Variation	0.72 ± 0.56
Network Normalized Duration IQR	0.67 ± 0.048
Synchrony Metrics and Average Network Burst Metrics	
Area Under Normalized Cross-Correlation	0.039 ± 0.003
Network Burst Peak Amplitude (max spikes per *sec*)	171.6 ± 11.1
n	102

10-minute (600 s) recording duration.

### 3.2 Naloxone reverses DAMGO-Induced electrophysiological effects

**Fig. 2:**
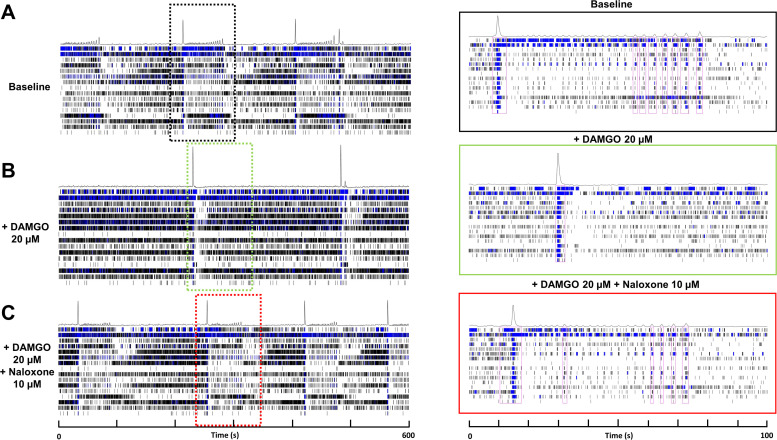
Neural Activity Response to DAMGO and naloxone Treatment. Example raster plots taken from well-treated with DAMGO [20 µM] for 90-minutes followed by treatment with naloxone [10 µM] for 30-minutes. A) Raster plot of 10-minute baseline recording (left panel) and detailed view of 100 s interval (black dotted box, right panel) demonstrating spontaneous spiking and bursting activity. B) Raster plot following addition of DAMGO [20 µM] (left panel) and 100 s detailed view (green dotted box, right panel) demonstrating increased individual spiking and reduced network burst. C) Raster plot of naloxone [10 µM] addition with corresponding 100 s interval view (red dotted box, right panel) demonstrating reversal of individual spiking and network bursting activity.

**Fig. 3:**
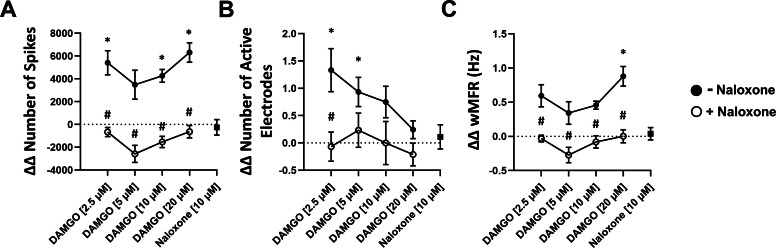
Electrophysiological Effects of DAMGO Treatment on Spiking Activity Metrics. Neural NAM electrophysiological activity resulting from exposure to DAMGO concentrations 2.5 µM, 5 µM, 10 µM, 20 µM (filled circles, following addition of 10 µM naloxone (open circles), and in response to 10 µM naloxone alone (filled square). Summary data graphs for A) ΔΔ Number of Spikes, B) ΔΔ Number of active electrodes, and C) ΔΔ weighted mean firing rate (wMFR). Data are mean ± SEM. *n* = 9 to 12 per condition. Vehicle vs DAMGO (*p-value < 0.05); naloxone [10 µM] + DAMGO vs DAMGO (#p-value < 0.05).

**Fig. 4:**
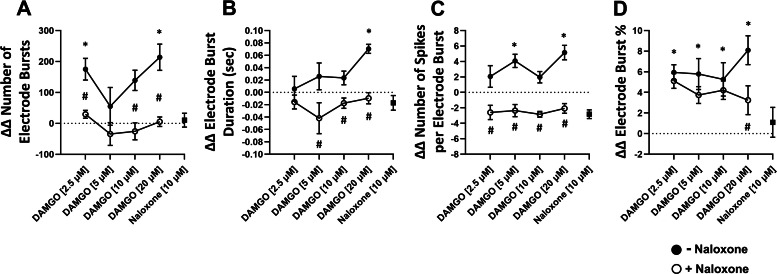
Electrophysiological Effects of DAMGO Treatment on Single Electrode Burst Metrics. Neural NAM electrophysiological activity resulting from exposure to DAMGO concentrations 2.5 µM, 5 µM, 10 µM, 20 µM (filled circles, following addition of 10 µM naloxone (open circles), and in response to 10 µM naloxone alone (filled square). Summary data graphs for A) ΔΔ Number of electrode bursts, B) ΔΔ Electrode burst duration, C) ΔΔ Number of spikes per electrode burst, and D) ΔΔ Electrode burst %. Data are mean ± SEM. *n* = 9 to 12 per condition. Vehicle vs DAMGO (*p-value < 0.05); naloxone [10 µM] + DAMGO vs DAMGO (#p-value < 0.05).

**Fig. 5:**
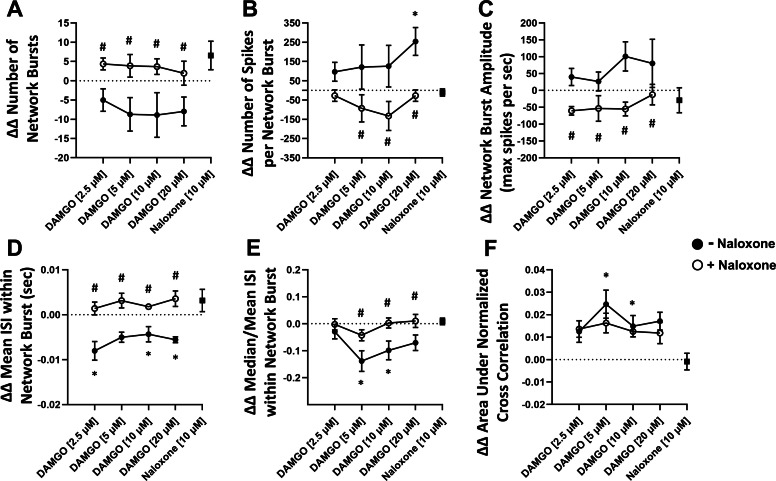
Electrophysiological Effects of DAMGO Treatment on Network and Synchrony Metrics. Neural NAM electrophysiological activity resulting from exposure to DAMGO concentrations 2.5 µM, 5 µM, 10 µM, 20 µM (filled circles, following addition of 10 µM naloxone (open circles), and in response to 10 µM naloxone alone (filled square). Summary data graphs for A) ΔΔ Number of network bursts, B) ΔΔ Number of spikes per network burst, C) ΔΔ Network burst amplitude, D) ΔΔ Mean ISI within network burst, E) Median/Mean ISI within network burst, and F) ΔΔ Area under normalized cross correlation. Data are mean ± SEM. *n* = 9 to 12 per condition. Vehicle vs DAMGO (*p-value < 0.05); naloxone [10 µM] + DAMGO vs DAMGO (#p-value < 0.05).

To assess the effects of DAMGO on human neural electrophysiology, we evaluated electrophysiological metrics following 90 min DAMGO treatment and 30 min naloxone reversal (Fig. 2). We found that DAMGO treatment resulted in significant changes in individual spiking activity including significantly increased spike number and weighted mean firing rate in a concentration-dependent manner relative to vehicle control (Fig. 3A,C). DAMGO treatment significantly increased the number of active electrodes at 2.5 µM and returned to comparable levels as vehicle control at concentrations above 10 µM (Fig. 3B). Likewise, DAMGO induced significant changes in single electrode activity including increased electrode burst number, duration, and spikes per single electrode burst (Fig. 4A,C). Moreover, DAMGO treatment increased the number of total spikes occurring within single electrode burst along with increased electrode burst percentage relative to vehicle control (Fig. 4D). On the other hand, DAMGO treatment resulted in a modest but nonsignificant concentration-dependent reduction of the network burst number relative to vehicle control (Fig. 5A). DAMGO treatment resulted in an increase in the number of spikes per network burst and a shortened inter spike interval within the network burst (Fig. 5B,D). Additionally, median/mean ISI within the burst was reduced suggesting less organized spikes within the network burst. The DAMGO treatment increased neural synchrony as early as 5 µM. These results suggest that DAMGO induces differential effects on individual, single electrode (i.e., local) and well wide global neural electrophysiological activity in vitro. Addition of the opioid antagonist, naloxone 10 µM, reversed the DAMGO-induced effects following 30 min incubation. Moreover, the effects of DAMGO treatment on network burst number and burst amplitude did not reach statistical significance, when evaluating the population response, relative to vehicle control. We did observe a significant difference between DAMGO alone relative to DAMGO when combined with naloxone (number of network burst, *P* = 0.0006) (Fig. 5A) suggesting antagonist-induced effects. Evaluation of a naloxone only control revealed modest nonsignificant effects on various neural parameters relative to vehicle control at the concentration used in this study. These data demonstrate that the neural NAM responds to the opioid DAMGO in a concentration-dependent manner and that these effects are reversed by the opioid antagonist naloxone in vitro.

### 3.3 DAMGO disrupts baseline network electrophysiological patterns

**Fig. 6:**
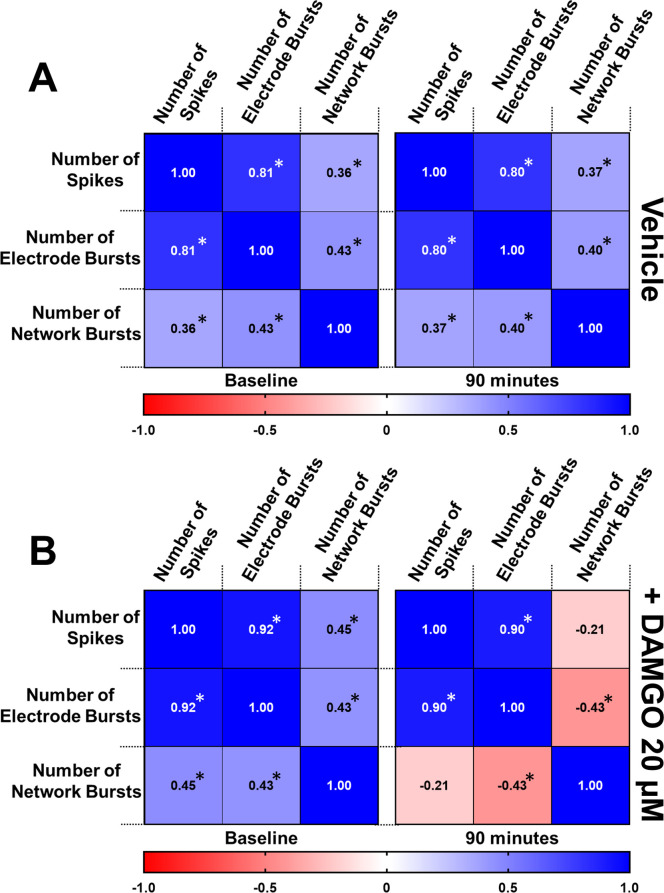
Correlation matrix displaying Pearson r values between neural electrophysiological parameters spikes, electrode bursts, and network bursts at baseline (left) and 90 min following A) vehicle and B) DAMGO 20 µM treatment (right). Positive correlations shown in blue, negative correlations shown in red. (*p-value < 0.05).

To investigate the relationship between neural metrics at baseline and following exposure to DAMGO, we performed a correlation analysis. Three neural activity patterns have been included to illustrate the functional relationship between neuronal spikes, single electrode bursts, and higher order network bursting activity. At baseline, the neural NAM displayed a strong positive and significant correlation between neuronal spiking and single electrode bursts (Fig. 6A). Likewise, a weaker but significant positive correlation was established between neuronal spiking and global network bursting (Fig. 6A). Similarly, single electrode bursts and global network bursts also displayed a significant positive correlation (Fig. 6A). Notably, 90 min of exposure to the vehicle control displayed negligible effects on the established baseline neural electrical activity relationships. (Fig. 6A). Having identified correlations between baseline neural metrics, we next evaluated the effects of 20 µM DAMGO on these baseline neural patterns. We found that 90 min of exposure to DAMGO resulted in significant effects on various neural patterns (Fig. 6B, Supplemental Table 1). Interestingly, DAMGO treatment had negligible effect on the significant positive correlation between neuronal spiking and single electrode bursts established at baseline (Fig. 6B). Whereas DAMGO treatment inhibited higher order connectivity such that the significant positive correlation between neuronal spiking and network bursts was reversed and instead established a negative correlation (Fig. 6B). To an even greater degree, treatment with DAMGO resulted in a complete inversion of the significant positive correlation between single electrode bursts and global network bursts (Fig. 6B) observed at baseline. These results suggest that DAMGO eliminates higher order neural network activity independent of spike number and single electrode bursts in a human neural NAM.

### 3.4 Baseline network activities modulate DAMGO network response

**Fig. 7:**
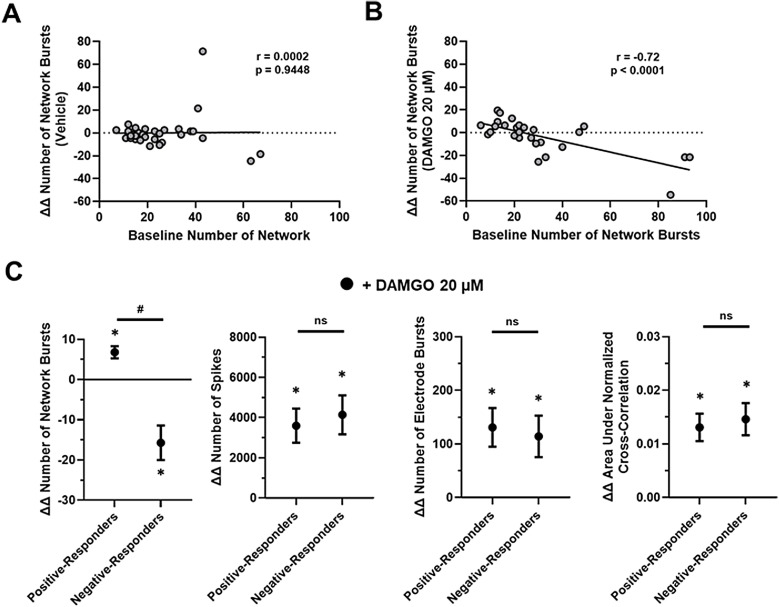
Linear Regression and Subpopulation Analysis. Linear regression analysis comparing the baseline number of network bursts and ΔΔ number of network bursts following treatment with A) vehicle and B) DAMGO 20 µM. C) Summary data graphs of neural NAM electrophysiological activity for positive and negative responders following DAMGO 20 µM treatment for ΔΔ number of network bursts, ΔΔ number of spikes, ΔΔ number of electrode bursts, and ΔΔ area under normalized cross correlation. Data are mean ± SEM. *n* = 12 to 15 per condition. Vehicle vs Positive-Responders or Vehicle vs Negative-Responders (*p-value < 0.05); Positive-Responders vs Negative-Responders (#p-value < 0.05).

Given that baseline network activity has been shown to correlate with opioid-induced response, we applied a linear regression analysis and found a significant negative linear relationship between the baseline number of network bursts and the DAMGO induced network response (ΔΔ Number of Network Burst) after 90 min (Fig. 7B). Comparing all baseline parameters to the DAMGO induced network response (ΔΔ Number of Network Burst) revealed this pairing also held the strongest correlation (Supplemental Table 2). In contrast, the vehicle control was not linearly related (Fig. 7A) and displayed no correlation. Because opioid exposure has been shown to have variable effects on neural activity, we quantified DAMGO response across individual wells. As a starting point, we stratified networks into subpopulations by the reproducible divergent DAMGO induced network response relative to that of vehicle control (Incognito et al., 2018). "Positive-responders" were defined as those with an above zero DAMGO induced number of network bursts (ΔΔ Number of Network Burst), while networks with a below zero number of induced network bursts were considered "negative-responders". As expected, negative-responder networks displayed a significant DAMGO-induced reduction in network burst number relative to vehicle control (Fig. 7C). Whereas the opposite was observed in positive-responder networks which displayed a significant increase in network burst number (Fig. 7C) relative to vehicle control. Moreover, while positive and negative responders both displayed an increase in spike number, single electrode burst number, and synchrony relative to vehicle control, there was no significant difference in neural spiking parameters and single electrode burst parameters between the groups following DAMGO treatment (Supplemental Table 3). On the other hand, the negative responder group displayed significant differences in higher order network burst organization including duration, spikes within the burst, and spike distribution relative to the positive responders. Additionally, network burst amplitude was significantly increased in the negative responder group while unchanged in the positive group relative to vehicle control (Supplemental Table 3). To understand this divergent response, we evaluated the baseline network electrophysiological properties of both positive and negative responder groups and identified a significant difference in number of network bursts at baseline. Such that the negative-responders displayed a significantly increased mean baseline network burst number relative to the positive responders of 42.5 ± 8.5 and 21.07 ± 3.2 (*P* = 0.02), respectively. To test whether the DAMGO-induced effects on network burst number (ΔΔ Number of Network Burst) is indeed influenced by the baseline network activity we stratified networks by varying degree of baseline excitability (i.e., activity) (Chou et al., 2024). "High-activity" networks were defined as those with an above mean (*X* = 25.7 ± 1.7) (Table 1) baseline network burst number, while networks with a below mean baseline network burst number were considered "low-activity". For high-activity networks DAMGO treatment resulted in a mean ΔΔ number of network burst of −18.30 ± 4.7 (*P* = 0.0527) relative to vehicle control. Conversely, low-activity networks displayed a mean of 7.7 ± 1.8 (*P* = 0.0003). Taken together; these findings suggest that the baseline network activity contributes to DAMGO induced network response in neural NAMs in vitro.

## 4 Discussion

### 4.1 Opioid induced effects in hiPSC neural NAM and naloxone reversal

In this study, we extend the application of a hiPSC neural MEA assay towards the evaluation of an opioid agonist and opioid antagonist combination in vitro. While analogues of this assay have been previously validated for use in assessing a variety of CNS active compounds, there is considerable heterogeneity between the cellular compositions used across the studies from various commercial providers and academic laboratories (Ishibashi et al., 2023; Nimbalkar et al., 2023; A.M. Tukker et al., 2020; Tukker et al., 2018). Growing evidence shows that to achieve an appropriate degree of neuronal excitatory-inhibitory balance and stability, it is critical an adequate representation of the supportive glial population is also present (A.M. Tukker et al., 2020). As such we adapted a mixed neuronal-glial co-culture platform. In opting for an isogenic co-culture approach, we selected commercially available hiPSC neurons and hiPSC astrocytes from the same donor for integration into the NAM. To our knowledge, a detailed evaluation of the effects of opioid reversal has not been conducted for this model. We demonstrated that DAMGO treatment markedly modulated individual, local, and global neural electrophysiological activity, in a concentration-dependent manner, in a neural NAM utilizing cells derived from a “healthy” donor. Importantly, we show that these effects are reversed by addition of the opioid antagonist naloxone. Moreover, we demonstrate opioid-induced disruption of higher order network synaptic communication relative to baseline and vehicle control. These findings demonstrate that nonclinical neural NAMs including hiPSC neural cells combined with MEA can be used to evaluate electrophysiological changes induced and reversed by opioid receptor modulators in a high-throughput format.

### 4.2 Comparison of nonclinical opioid studies

Lacking access to human primary neural cells is a common gap for the field. Recent commercialization of hiPSC-derived neurons has rapidly increased their availability and application further advancing our understanding of their context-of-use relative to rat primary cortical cultures and brain slices as the gold standard. However, much of our foundational knowledge of the CNS physiology has been learned from nonclinical animal studies. For example, in vitro and in vivo models composed of primarily glutamatergic neuron populations have demonstrated that exposure to the opioid agonist DAMGO typically results in a concentration-dependent reduction in excitatory behavior, particularly in higher order activity (Burgraff et al., 2021; Gumnit et al., 2022; Langer et al., 2017; Sun et al., 2019; Wei and Ramirez, 2019). These findings are consistent with recent studies utilizing subtype specific hiPSC-neurons demonstrating robust DAMGO-induced reduction of action potential frequency and naloxone reversal, based on lower throughput patch clamp studies (Guo et al., 2024). Likewise, a study employing complex 3D neural spheres reported reduced calcium oscillation frequency following 60 min DAMGO treatment including naloxone reversal (Strong et al., 2023), based on an indirect second-messenger calcium readout, in a high-throughput format. Here, we leverage these important studies and expand them to a direct neuronal electrophysiological readout including noninvasive high-throughput capabilities. Indeed, when introduced to the opioid agonist we observed an overall opioid-induced disruption of higher order network synaptic communication relative to baseline and vehicle control. Likewise, principal component analysis (PCA) was used to analyze the MEA drug response data (Supplemental Figure 2) (Supplemental Table 5). We observed distinct separation between DAMGO [20 µM] and vehicle treatment groups. While vehicle and DAMGO [20 µM] plus naloxone [10 µM] clustered together. Evaluation of factor loading values for PC1 revealed which MEA parameters (i.e., factors) contributed the most to this component. Although various parameters contributed the strongest parameters for individual spiking activity were number of spikes and weighted mean firing rate. For single electrode activity parameters include burst percentage and number of spikes per burst. For higher order network activity parameters include number of spikes per network burst per channel, number of spikes per network burst, median ISI within network burst, mean ISI within network burst, median/mean ISI within network burst and the synchrony parameter area under normalized cross-correlation. Of note, upon further examination we identified markedly reproducible variability in opioid responsiveness across the treated wells suggesting that neural in vitro systems comprised of the same cell population develop to express differing interaction-based mechanisms (Chou et al., 2024). Interestingly, this degree of variability has been reported throughout a multitude of opioid related in vitro studies yet remains a seemingly overlooked property despite being reminiscent of the variations observed in vivo and clinically (Baertsch et al., 2021; Levran et al., 2013; Ramirez et al., 2021; Ren et al., 2015). As such, we quantified this variability across all concentrations evaluated by our neural NAM assay and discovered DAMGO treatment decreased number of network bursts for 68 % of the wells and increased number of network bursts for 32 % of wells. Despite this divergent response, the mean effect was a concentration-dependent decrease in network burst number with increasing concentrations of DAMGO resembling previously reported behavior (Chou et al., 2024). Moreover, this relationship suggests networks with higher baseline network burst values had a greater magnitude reduction comparable to previous reports (Palkovic et al., 2023). The correlation data and subpopulation analysis we present offers additional insight towards explaining this phenomenon. As previously stated, there was no change in the relationship between the number of spikes and single electrode bursts following DAMGO treatment though their respective relationship with network bursts was effectively inverted (Fig. 6B). A behavior not observed with the vehicle treated group (Fig. 6A). These results suggest that at baseline, increases in one parameter proportionally translate into increases in the others. As such, it can be expected that an increase in spiking activity will additionally result in an increase in electrode bursts activity but ultimately translate into a decrease in network burst activity when treated with DAMGO. These results suggest that an increase in lower order activity elicited by DAMGO can cause a disruption in higher order connectivity as reflected by the resulting decrease in network burst values in a human neural NAM. While it is clear through our investigation that pre-existing relationships between our three defined categorical measures of communication (i.e. spikes, bursts, and network bursts) can be used to assess disruption caused by DAMGO, further analysis using differing drug classes is necessary to determine if this combinatorial approach can be used for safety assessment.

### 4.3 Potential regulatory considerations

An important take-home message is while the number of regulatory submissions including NAMs data is expected to increase in accordance with the FDA Roadmap 2025 and FDA Modernization act 2.0 (Congress, 2022; FDA, 2025). The utility of such data to assist regulatory decision making will depend on the specific context-of-use, quality, and validity of the data. As such, the CDER Drug Development Tool (DDT) qualification program specifically through the Innovative Science and Technology Approaches for New Drugs (ISTAND) pilot program accepts NAMs submissions to advance our understanding of drug safety and efficacy. However, a pathway for the validation and qualification for NAMs alternatives to animal studies has not been established yet by the FDA. It is expected that such a pathway will evolve as the FDA receives submissions of this information and gains more experience with techniques such as the one described herein.

### 4.4 Study limitations

Our study has several limitations. For example, while DAMGO is highly selective (Lindqvist et al., 2013) and readily available as a research tool it is not used in the clinical setting. As such, evaluation of additional opioids and illicit pharmacology are needed to enhance the translatability of our findings. The DAMGO concentration range selected in this study was determined based on previous hiPSC neural models (Strong et al., 2023) and the reported IC50 (Guo et al., 2024). However, it is plausible that higher DAMGO concentrations (e.g., 200 μM) may be needed to elucidate maximum effects based on the concentrations previously tested in vivo (Supplemental Table 4). Similarly, we did not investigate the reversibility of DAMGO-induced effects following washout in the neural NAM. The predetermined 90 min timepoint was based on previous nonclinical studies (Strong et al., 2023), the opioid overdose timescale (1 to 3 h), and the typical µ-opioid receptor internalization/desensitization time profile (Ma et al., 2020). In addition, molecular expression of the µ-opioid receptor in the hiPSC neurons used in our experiments have been reported elsewhere (Belair et al., 2025) and are not investigated here. Herein, we evaluated the effects of DAMGO on human neuronal electrophysiological properties. Additional acute and chronic timepoints are of interest but out of scope of the current study. Likewise, only a single concentration of naloxone was evaluated to reverse agonist induced effects limiting our ability to characterize naloxone antagonism including the minimum effective concentration in the neural NAM. Extension to additional opioid receptor antagonist and concentrations will aid data interpretations, comparisons, and clinical relevance. Furthermore, we observe a naloxone-induced increase in number of network burst (Supplemental Figure 3) at a higher concentration (i.e., 30 µM) however detailed analysis of endogenous opioids in the neural NAM may be needed to elucidate the mechanisms. While not evaluated here, previous nonclinical neural models have demonstrated functional dependance on extracellular potassium concentrations such that artificially increased extracellular potassium concentrations enhance neural activity at baseline (Bacak et al., 2016; Baertsch et al., 2021; Burgraff et al., 2021; Kallurkar et al., 2020; Mellen et al., 2003; Sun et al., 2019). However, in our study a lower (physiological) extracellular potassium concentration was utilized. Current commercially available MEA data analysis software programs do not enable robust assessment of neural burstlet characteristics including network burst inter-burst-interval, as such proprietary or laboratory specific custom-made software are routinely used but not readily available. In addition, future studies should consider well-exposure analysis to determine the amount of nonspecific binding in the human neural NAM. Finally, while we observe similarities between our findings and those from specific brain regions contributing to opioid-induced respiratory depression (OIRD). Such as the preBötzinger complex (preBötC), Bötzinger complex (BötC), and pontine we are not suggesting this model represents any specific region. Rather we demonstrate that a commercially available human cortical excitatory and inhibitory hiPSC neural NAM displays robust and reproducible response to opioid agonist and antagonist in vitro which may be useful for regulatory decision-making and in silico modeling.

## 5 Conclusion

This study provides a foundation for the nonclinical evaluation of opioid agonist and antagonist on human neuronal electrophysiological activity to support safety and efficacy assessment. Herein, we demonstrate several notable findings. 1) The hiPSC neural MEA assay responds to opioid agonist displaying DAMGO-dependent changes in electrical activity. 2) The observed changes are reversed by the opioid antagonist naloxone. 3) DAMGO treatment disrupts higher order synaptic network connectivity independent of neural spiking and electrode burst. 4) DAMGO-induced effects on network burst number are in part mediated by the hiPSC neural NAM baseline properties. 5) hiPSC neural NAM culture functionality develops over time. 6) Along with robust baseline neural network patterns. Future studies may evaluate the effects of additional opioid agonist and antagonist as well as the functional consequences of polypharmacy on the in vitro electrophysiological response to assist regulatory decision-making.

## Abbreviations

**Table:** 

NAMs	New approach methodologies
MPS	Microphysiological systems
PEI	Polyethylenimine
DAMGO	D-Ala(2)-mephe(4)-gly-ol(5))enkephalin
MEA	Multielectrode array
Cmax	Maximum drug concentration in serum
cBPM	Complete BrainPhys medium

## Author disclaimer

This article reflects the views of the author and should not be construed to represent FDA’s views or policies.

## Funding

This research was funded through the U.S. Food and Drug Administration.

## Publishers note

This publication reflects the views of the authors and should not be construed to represent FDA’s views or policies. The mention of commercial products, their sources, or their use in connection with material reported herein is not to be construed as either an actual or implied endorsement of such products by the Department of Health and Human Services.

## Generative AI statement

The authors declare that no Generative AI was used in the creation of this manuscript.

## CRediT authorship contribution statement

**Carlos Serna:** Writing – review & editing, Writing – original draft, Methodology, Investigation, Formal analysis, Data curation, Conceptualization. **Bhavya Bhardwaj:** Writing – review & editing, Investigation, Formal analysis, Data curation. **Tromondae K. Feaster:** Writing – review & editing, Writing – original draft, Visualization, Methodology, Investigation, Formal analysis, Data curation, Conceptualization. **Ksenia Blinova:** Writing – review & editing, Visualization, Supervision, Resources, Methodology, Funding acquisition, Conceptualization.

## Declaration of competing interests

The authors declare that they have no known competing financial interests or personal relationships that could have appeared to influence the work reported in this paper.

## Data Availability

The original contributions presented in the study are included in the article/supplementary material and can be found here: https://doi.org/10.6084/m9.figshare.30442544.v2. Further inquiries can be directed to the corresponding authors.

## Appendix Supplementary materials

Image, image 1

Image, application 2

Image, application 3

Image, application 4
