# Supplementary figures and images for: Nonclinical human neural new approach methodologies (NAMs): Electrophysiological assessment of opioid agonist and antagonist combination
Source: NAM J. 2025 Oct 22;1:100064. doi: 10.1016/j.namjnl.2025.100064 (PMC12856570; doi:10.1016/j.namjnl.2025.100064)

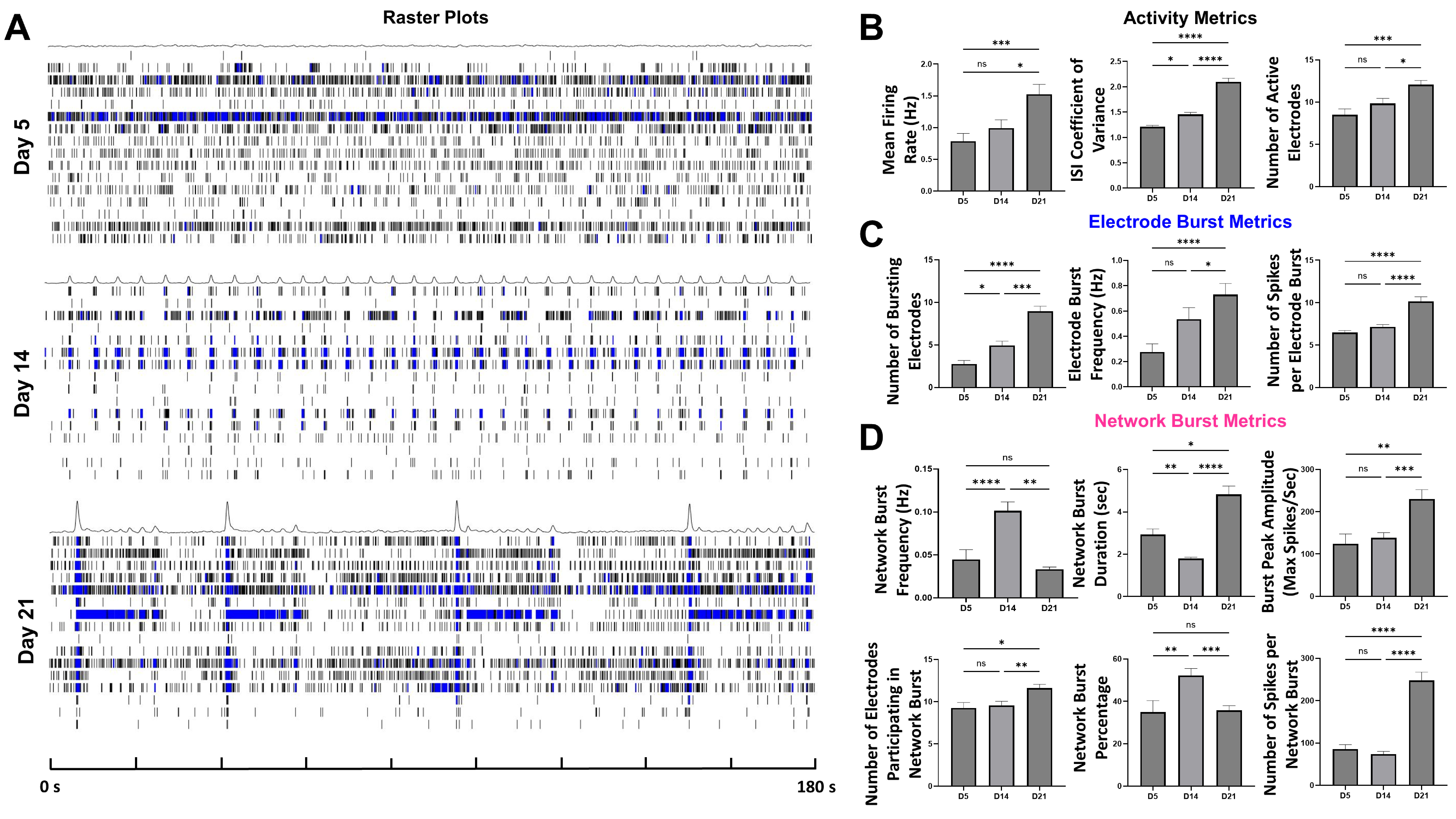

Supplement: Supplementary file 1 [file mmc1.jpg]

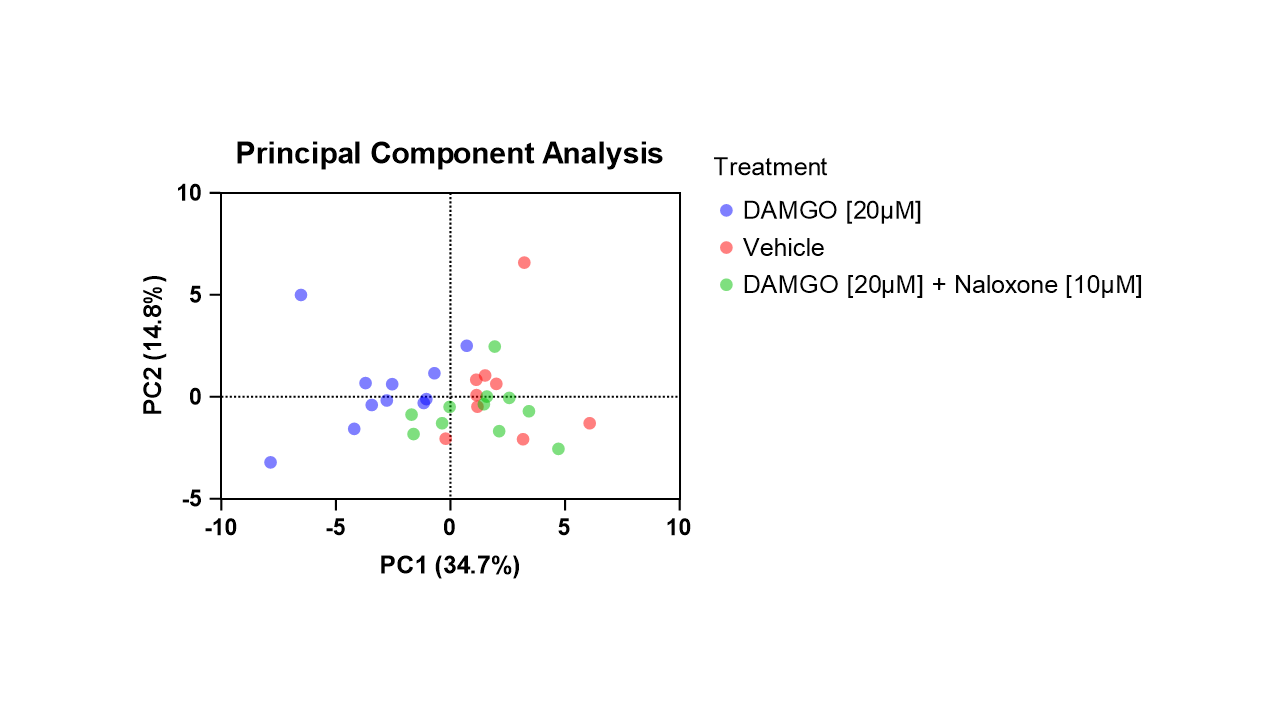

Supplement: Supplementary file 2 [file mmc2.zip › mmc2.tif]

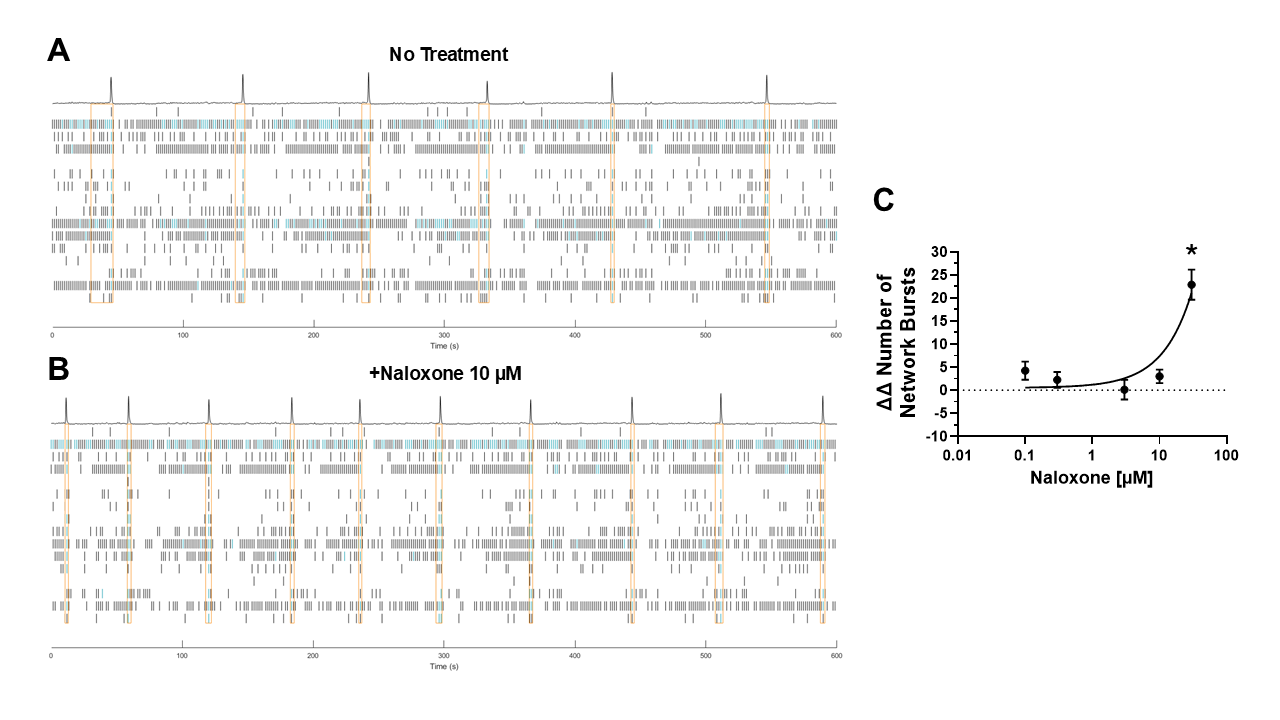

Supplement: Supplementary file 2 [file mmc2.zip › mmc3.tif]
