# Supplementary material for: Nonclinical human neural new approach methodologies (NAMs): Electrophysiological assessment of opioid agonist and antagonist combination
Source: NAM J. 2025 Oct 22;1:100064. doi: 10.1016/j.namjnl.2025.100064 (PMC12856570; doi:10.1016/j.namjnl.2025.100064)
Supplement: Supplementary file 3 [file mmc3.docx]

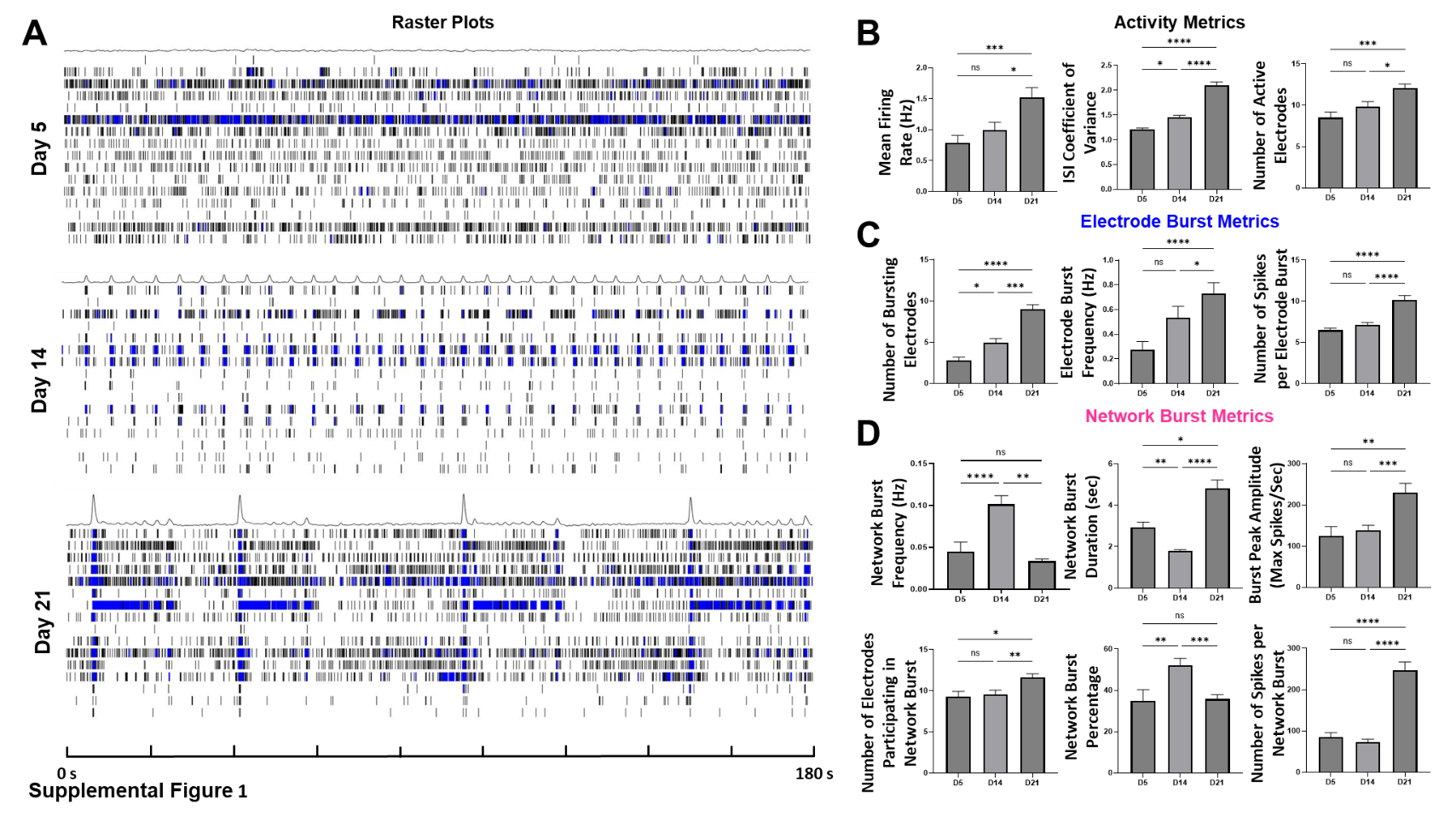
**Supplemental Material**

**Supplemental Figure 1.** Longitudinal Electrophysiological Activity. Neural activity recorded over a period of 180 seconds from days 5, 14 and 21 of co-culture system. A) Raster plots showing developing neuronal signaling throughout days 5, 14, and 21. B) Spiking activity metrics, quantifying degree of spiking throughout recording timeframe. C) Single electrode burst metrics, illustrating bursting behavior of spikes detected by MEA electrodes. D) Network burst metrics of neuronal network communication across all MEA electrodes in a single well. Summary data graphs are mean ± SEM, n = 30 per condition, *p-value < 0.05.

**Supplemental Figure 2.** Principal component analysis of MEA parameters. PCA data represented as a scatter plot incorporating 26 MEA parameters. Plot of first and second principal components (PC1 and PC2) for MEA data comparing Vehicle, DAMGO [20 µM], and DAMGO [20 µM] + naloxone [10 µM].


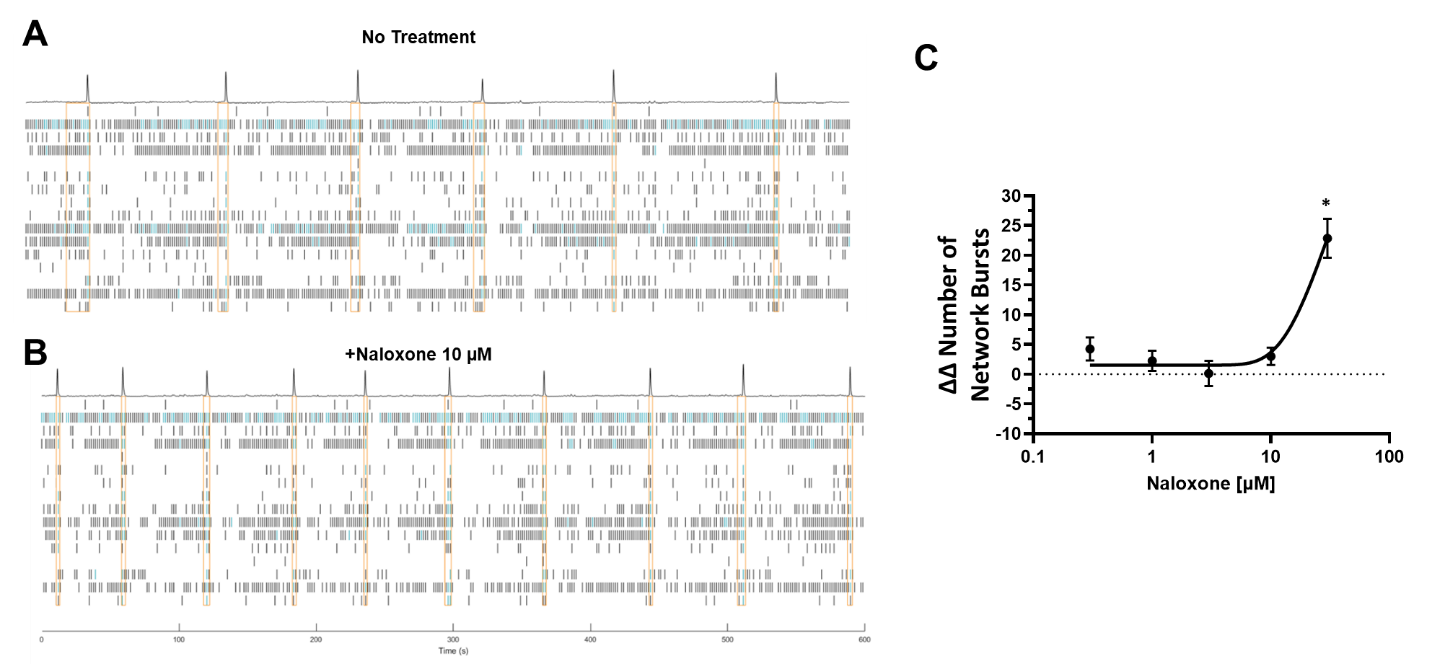


**Supplemental Figure 3.** Naloxone effects on neural activity. A) Raster plot of 10-minute no treatment recording. (B) Raster plot 30 minutes following addition of Naloxone [10 µM] demonstrating modest nonsignificant increase in number of network bursts. C) Summary data graph for naloxone concentration-response (ΔΔ Number of network bursts) following 30 minute exposure at concentrations 0.3 µM, 1 µM, 3 µM, 10 µM, 30 µM. At a concentration of [30 µM] Naloxone treatment significantly increased number of network bursts relative to baseline and vehicle control. Data are mean ± SEM. n = 8 per condition, DIV 31. Vehicle vs naloxone (*p-value < 0.05).

**
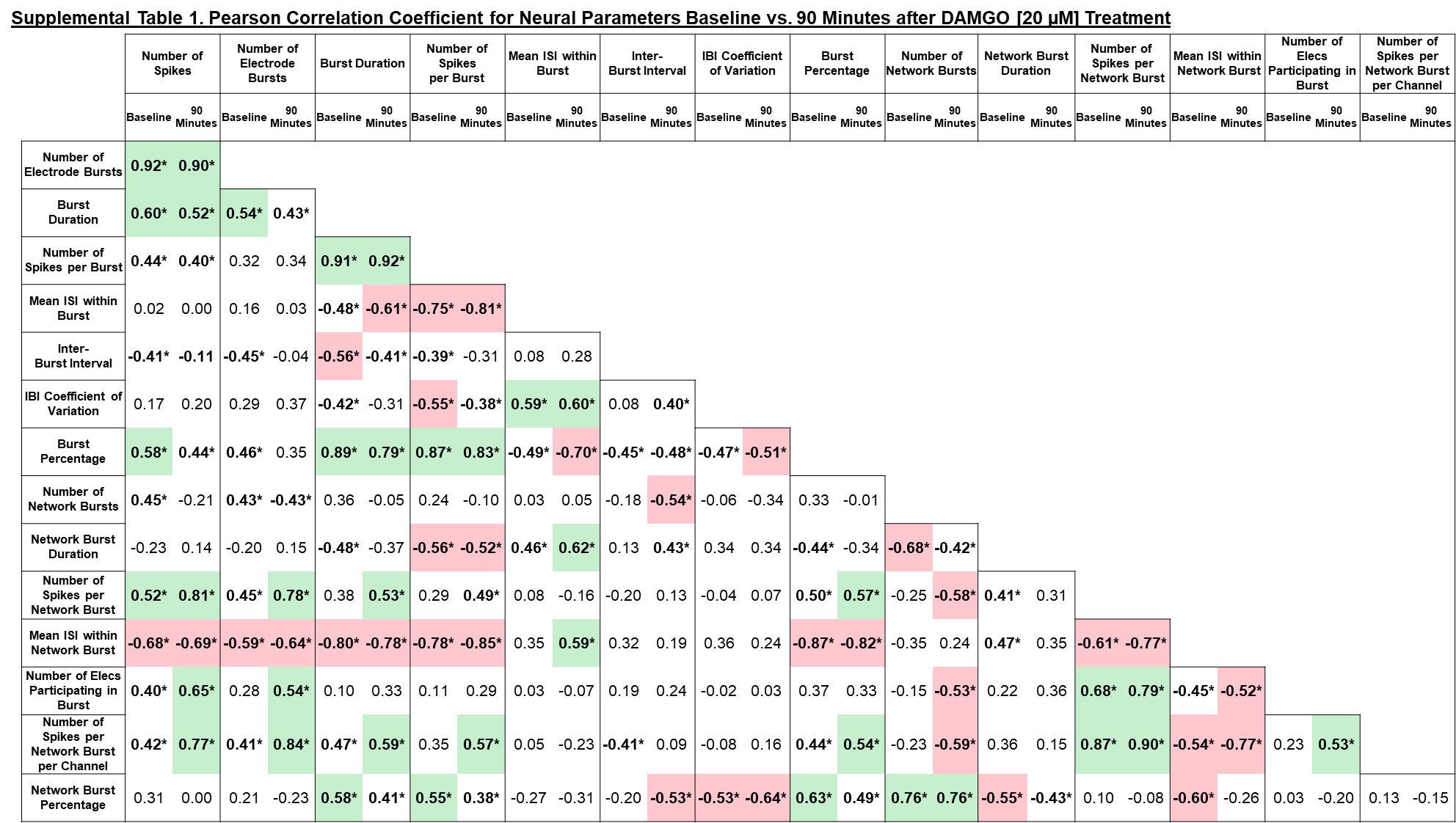
**

| **Supplemental Table 2. DAMGO [20 µM] ΔΔ Number of Network Burst Correlation vs Baseline Parameters** | |
| --- | --- |
|  |  |
| **Parameter** | **Pearson Correlation Coefficient (r)** |
| **Activity Metrics** |  |
| **Number of Spikes** | **-0.58** |
| **Mean Firing Rate (Hz)** | **-0.58** |
| **ISI Coefficient of Variation** | **-0.36** |
| **Number of Active Electrodes** | **-0.21** |
| **Weighted Mean Firing Rate (Hz)** | **-0.45** |
| **Electrode Burst Metrics** |  |
| **Number of Bursts** | **-0.6** |
| **Number of Bursting Electrodes** | **-0.15** |
| **Burst Duration (sec)** | **-0.44** |
| **Number of Spikes per Burst** | **-0.41** |
| **Mean ISI within Burst (sec)** | **0.04** |
| **Median ISI within Burst (sec)** | **0.00** |
| **Median/Mean ISI within Burst** | **-0.03** |
| **Inter-Burst Interval (sec)** | **-0.05** |
| **IBI Coefficient of Variation** | **0.12** |
| **Burst Percentage** | **-0.45** |
| **Network Burst Metrics** |  |
| **Number of Network Bursts** | **-0.72** |
| **Network Burst Duration (sec)** | **0.44** |
| **Number of Spikes per Network Burst** | **0.09** |
| **Mean ISI within Network Burst (sec)** | **0.40** |
| **Median ISI within Network Burst (sec)** | **0.28** |
| **Median/Mean ISI within Network Burst** | **-0.13** |
| **Number of Elecs Participating in Burst** | **-0.12** |
| **Number of Spikes per Network Burst per Channel** | **0.17** |
| **Network Burst Percentage** | **-0.55** |
| **Network IBI Coefficient of Variation** | **-0.07** |
| **Network Normalized Duration IQR** | **0.45** |
| **Synchrony Metrics and Average Network Burst Metrics** |  |
| **Area Under Normalized Cross-Correlation** | **-0.27** |
| **Burst Amplitude Peak (Max Spikes per sec** | **0.15** |
| **Time to Burst Peak (ms)** | **-0.29** |

| **Supplemental Table 3. Positive and Negative Responder Analysis of Electrophysiological Properties Following DAMGO [20 µM] Treatment** | | | | | |
| --- | --- | --- | --- | --- | --- |
|  | **Positive Responders vs Vehicle** | | **Negative Responders vs Vehicle** | | **Positive Responders vs Negative Responders** |
| **Parameter** | **Mean ± SEM** | **P Value** | **Mean ± SEM** | **P Value** | **P Value** |
| **Activity Metrics** |  |  |  |  |  |
| **ΔΔ Number of Spikes** | **3599.9 ± 848.4** | **<0.0001*** | **4141.3 ± 971.1** | **<0.0001*** | **0.2995** |
| **ΔΔ Mean Firing Rate (Hz)** | **0.38 ± 0.1** | **<0.0001*** | **0.43 ± 0.1** | **<0.0001*** | **0.2995** |
| **ΔΔ ISI Coefficient of Variation** | **0.31 ± 0.1** | **<0.0001*** | **0.18 ± 0.1** | **0.0368*** | **0.1971** |
| **ΔΔ Number of Active Electrodes** | **-0.21 ± 0.2** | **0.2447** | **-0.02 ± 0.2** | **0.9887** | **0.3295** |
| **ΔΔ Weighted Mean Firing Rate (Hz)** | **0.68 ± 0.2** | **<0.0001*** | **0.65 ± 0.1** | **<0.0001*** | **0.9809** |
| **Electrode Burst Metrics** |  |  |  |  |  |
| **ΔΔ Number of Electrode Bursts** | **130.9 ± 36.1** | **0.0003*** | **114.1 ± 38.8** | **0.0028*** | **>0.9999** |
| **ΔΔ Number of Bursting Electrodes** | **0.9 ± 0.3** | **0.0032*** | **0.8 ± 0.2** | **0.0029*** | **0.8141** |
| **ΔΔ Burst Duration (sec)** | **0.03 ± 0.01** | **0.0038*** | **0.1 ± 0.01** | **<0.0001*** | **0.0999** |
| **ΔΔ Number of Spikes per Burst** | **2.28 ± 0.5** | **0.0003*** | **4.2 ± 1.1** | **<0.0001*** | **0.0901** |
| **ΔΔ Mean ISI within Burst (sec)** | **0.001 ± 0.001** | **0.8565** | **-0.001 ± 0.001** | **0.3127** | **0.3684** |
| **ΔΔ Median ISI within Burst (sec)** | **0.001 ± 0.001** | **0.7581** | **-0.001 ± 0.001** | **0.2261** | **0.2181** |
| **ΔΔ Median/Mean ISI within Burst** | **0.01 ± 0.01** | **0.6921** | **-0.02 ± 0.01** | **0.0486*** | **0.0616** |
| **ΔΔ Inter-Burst Interval (sec)** | **-7.4 ± 7.6** | **0.3099** | **-19.9 ± 9.2** | **0.0832*** | **0.299** |
| **ΔΔ IBI Coefficient of Variation** | **-0.02 ± 0.1** | **0.9825** | **0.01 ± 0.04** | **0.9294** | **0.7112** |
| **Burst Percentage** | **8.04 ± 1.5** | **<0.0001*** | **6.3 ± 1.1** | **<0.0001*** | **0.8667** |
| **Network Burst Metrics** |  |  |  |  |  |
| **ΔΔ Number of Network Bursts** | **6.8 ± 1.5** | **<0.0001*** | **-15.7 ± 4.3** | **0.0003*** | **<0.0001*** |
| **ΔΔ Network Burst Duration (sec)** | **-1.62 ± 0.6** | **0.0298*** | **-0.03 ± 0.01** | **0.4389** | **0.0063*** |
| **ΔΔ Number of Spikes per Network Burst** | **29.7 ± 26.8** | **0.0195*** | **200.1 ± 65.9** | **<0.0001*** | **0.0017*** |
| **ΔΔ Mean ISI within Network Burst (sec)** | **-0.01 ± 0.001** | **<0.0001*** | **-0.01 ± 0.001** | **<0.0001*** | **0.9809** |
| **ΔΔ Median ISI within Network Burst (sec)** | **-0.003 ± 0.001** | **<0.0001*** | **-0.01 ± 0.001** | **<0.0001*** | **0.2363** |
| **ΔΔ Median/Mean ISI within Network Burst** | **-0.02 ± 0.02** | **0.2792** | **-0.1 ± 0.01** | **<0.0001*** | **0.0055*** |
| **ΔΔ Number of Elecs Participating in Burst** | **0.2 ± 0.1** | **0.3317** | **0.7 ± 0.2** | **0.0015*** | **0.0262*** |
| **ΔΔ Number of Spikes per Network Burst per Channel** | **2.04 ± 2.6** | **0.0208*** | **15.5 ± 4.0** | **<0.0001*** | **0.0009*** |
| **ΔΔ Network Burst Percentage** | **10.99 ± 1.7** | **0.0005*** | **-2.4 ± 2.4** | **0.4813** | **0.0001*** |
| **ΔΔ Network IBI Coefficient of Variation** | **-0.1 ± 0.1** | **0.6283** | **-0.3 ± 0.1** | **0.0786** | **0.3229** |
| **ΔΔ Network Normalized Duration IQR** | **0.1 ± 0.1** | **0.7415** | **0.1 ± 0.1** | **0.7894** | **0.9404** |
| **Synchrony Metrics and Average Network Burst Metrics** |  |  |  |  |  |
| **ΔΔ Area Under Normalized Cross-Correlation** | **0.01 ± 0.003** | **<0.0001*** | **0.01 ± 0.003** | **0.0002*** | **0.5806** |
| **ΔΔ Burst Amplitude Peak (Max Spikes per sec)** | **-5.07 ± 13.1** | **0.5854** | **86.5 ± 64.0** | **0.0486*** | **0.0469*** |
| **ΔΔ Time to Burst Peak (ms)** | **21.34 ± 9.2** | **0.0604** | **-22.1 ± 40.5** | **0.3984** | **0.0074*** |

| **Supplemental Table 4. Estimated DAMGO In vivo Exposure Assessment.** | | |
| --- | --- | --- |
| **Concentration (µM)** | **Species** | **Reference** |
| **200** | **rabbit** | [**PMID: 35124284**](https://pubmed.ncbi.nlm.nih.gov/35124284/) |
| **100** | **rabbit** | [**PMID: 34352068**](https://pubmed.ncbi.nlm.nih.gov/34352068/) |
| **10 - 100** | **goat** | [**PMID: 28137700**](https://pubmed.ncbi.nlm.nih.gov/28137700/) |
| **100** | **rabbit** | [**PMID: 28590302**](https://pubmed.ncbi.nlm.nih.gov/28590302/) |
| **100** | **rabbit** | [**PMID: 25751234**](https://pubmed.ncbi.nlm.nih.gov/25751234/) |
| **0.000059** | **rat** | [**PMID: 26175072**](https://pubmed.ncbi.nlm.nih.gov/26175072/) |
| **0.07 - 144.4** | **rat** | **PMID: 22672845** |
| **~0.3^** | **rat** | **PMID: 22934681** |
| **~1.5#** | **rat** | **PMID: 22934681** |
| **~9.7** | **rat** | **PMID: 22934681** |
| **100** | **canine** | **PMID: 22875901** |
| **5 - 200** | **rats** | [**PMID: 21273414**](https://pubmed.ncbi.nlm.nih.gov/21273414/) |
| **100** | **canine** | [**PMID: 19906886**](https://pubmed.ncbi.nlm.nih.gov/19906886/) |
| **~1.8** | **sheep** | **PMID: 11408525** |
| **# unbound in blood, ^unbound in brain** | | |

| **Supplemental Table 5. Principal components factor loading values for select MEA parameters analyzed** | | | |  |  |
| --- | --- | --- | --- | --- | --- |
| **Parameter** | **PC1** | **PC2** | **PC3** | |  |
| **Median ISI within Network Burst - Avg (sec)** | **0.770404556** | **-0.120402452** | **0.160459281** | |  |
| **Mean ISI within Network Burst - Avg (sec)** | **0.761141032** | **-0.284563713** | **0.295504237** | | |
| **Median/Mean ISI within Network Burst - Avg** | **0.75503637** | **0.287494864** | **-0.221160916** | | |
| **Network IBI Coefficient of Variation** | **0.559605501** | **-0.175736726** | **-0.174113809** | | |
| **Mean ISI within Burst - Avg (sec)** | **0.516649145** | **0.08497528** | **-0.677581414** | | |
| **Median ISI within Burst - Avg (sec)** | **0.430544761** | **0.131247103** | **-0.77425567** | | |
| **Inter-Burst Interval - Avg (sec)** | **0.328592527** | **-0.352937466** | **-0.47367917** | | |
| **Number of Network Bursts** | **0.297787635** | **0.859011606** | **-0.091175688** | | |
| **Median/Mean ISI within Burst - Avg** | **0.257445558** | **0.323941897** | **-0.64798595** | | |
| **IBI Coefficient of Variation - Avg** | **0.206148883** | **-0.481405883** | **-0.496770041** | | |
| **Time to Burst Peak (ms)** | **0.203860497** | **0.45491785** | **0.392449385** | | |
| **Network Burst Percentage** | **0.083389079** | **0.675611931** | **-0.330719785** | | |
| **Network Burst Duration - Avg (sec)** | **0.02098976** | **-0.4465318** | **-0.394264489** | | |
| **Number of Active Electrodes** | **-0.174401527** | **-0.263282557** | **-0.231838236** | | |
| **ISI Coefficient of Variation - Avg** | **-0.347256008** | **0.190523389** | **-0.166194802** | | |
| **Burst Frequency - Avg (Hz)** | **-0.414686961** | **0.697902615** | **-0.092216595** | | |
| **Burst Peak (Max Spikes per sec)** | **-0.474405277** | **-0.417920652** | **-0.036205788** | | |
| **Number of Bursting Electrodes** | **-0.538471043** | **-0.335450585** | **-0.179331035** | | |
| **Burst Duration - Avg (sec)** | **-0.682124644** | **-0.265398616** | **-0.192754993** | | |
| **Number of Elecs Participating in Burst - Avg** | **-0.703918261** | **-0.143970053** | **0.141871281** | | |
| **Number of Bursts** | **-0.745309524** | **0.37874213** | **-0.143051594** | | |
| **Area Under Normalized Cross-Correlation** | **-0.753776187** | **0.072419592** | **0.185014216** | | |
| **Weighted Mean Firing Rate (Hz)** | **-0.767344479** | **0.464070214** | **-0.189999933** | | |
| **Number of Spikes per Network Burst - Avg** | **-0.798855477** | **-0.310261526** | **-0.245797132** | | |
| **Number of Spikes per Burst - Avg** | **-0.821135894** | **-0.322283563** | **-0.032061872** | | |
| **Burst Percentage - Avg** | **-0.821362159** | **0.340344855** | **0.07676337** | | |
| **Number of Spikes per Network Burst per Channel - Avg** | **-0.823563934** | **-0.278540696** | **-0.29571329** | | |
| **Number of Spikes** | **-0.823794376** | **0.357451724** | **-0.254082429** | | |

**Parameters sorted by descending PC1 loading value.**
